# Assessing effects on dendritic arborization using novel Sholl analyses

**DOI:** 10.3389/fncel.2015.00285

**Published:** 2015-07-30

**Authors:** Kate M. O'Neill, Barbara F. Akum, Survandita T. Dhawan, Munjin Kwon, Christopher G. Langhammer, Bonnie L. Firestein

**Affiliations:** ^1^Department of Cell Biology and Neuroscience, Rutgers UniversityPiscataway, NJ, USA; ^2^Graduate Program in Biomedical Engineering, Rutgers UniversityPiscataway, NJ, USA

**Keywords:** neuron, dendrite, Sholl, morphology, tracing, image analysis

## Abstract

Determining the shape of cell-specific dendritic arbors is a tightly regulated process that occurs during development. When this regulation is aberrant, which occurs during disease or injury, alterations in dendritic shape result in changes to neural circuitry. There has been significant progress on characterizing extracellular and intrinsic factors that regulate dendrite number by our laboratory and others. Generally, changes to the dendritic arbor are assessed by Sholl analysis or simple dendrite counting. However, we have found that this general method often overlooks local changes to the arbor. Previously, we developed a program (titled Bonfire) to facilitate digitization of neurite morphology and subsequent Sholl analysis and to assess changes to root, intermediate, and terminal neurites. Here, we apply these different Sholl analyses, and a novel Sholl analysis, to uncover previously unknown changes to the dendritic arbor when we overexpress an important regulator of dendrite branching, cytosolic PSD-95 interactor (cypin), at two developmental time points. Our results suggest that standard Sholl analysis and simple dendrite counting are not sufficient for uncovering local changes to the dendritic arbor.

## Introduction

Neurons are polarized cells that send information through a main axon and receive information through highly branched dendrites. The development and patterning of dendrites is a tightly regulated process that is essential for proper functioning of the central nervous system. The overall shape of the dendritic arbor determines the inputs that neurons receive and how inputs are processed, thus affecting synaptic output (Miller and Jacobs, 1984; Eilers and Konnerth, 1997; Hausser et al., 2000; Vetter et al., 2001; Schaefer et al., 2003; Elston and Fujita, 2014). The arbor is shaped by intrinsic and extrinsic factors (Landgraf and Evers, 2005; Libersat, 2005; Santiago and Bashaw, 2014; Dong et al., 2015; Sainath and Gallo, 2015) and can also be influenced by trauma or disease (reviewed in Kulkarni and Firestein, 2012). Disorders in which neuronal morphology is disturbed highlight the importance of proper dendritic shape to the overall functioning of neuronal networks (Zoghbi, 2003; Kulkarni and Firestein, 2012).

A number of metrics may be used to identify dendritic arbor morphology (Uylings and Van Pelt, 2002). Sholl analysis (Sholl, 1953) has been an instrumental tool in revealing changes to the dendritic arbor as a whole. Sholl analysis includes counting the number of dendritic intersections that occur at fixed distances from the soma in concentric circles. This analysis reveals the number of branches, branch geometry, and overall branching patterns of neurons (Caserta et al., 1995). Performing this process by hand is time-consuming and introduces inherent variability due to inconsistency and experimenter bias. Our laboratory developed a semi-automated Sholl analysis program, called Bonfire, that not only performs analysis on the entire arbor but also analyzes subsets of dendrites (primary/secondary/tertiary, root/intermediate/terminal) within the arbor (Kutzing et al., 2010; Langhammer et al., 2010). This detailed reporting of the data allows for morphological analysis to occur on a much smaller scale.

A major focus of our work is to understand how changes to the dendritic arbor are mediated by various intrinsic and extrinsic factors. We identified a protein termed cypin (*cy*tosolic *P*SD-95 *in*teractor) as a core regulator of dendritic arborization (Akum et al., 2004; Fernandez et al., 2008). Cypin promotes local microtubule assembly in the dendrite by binding tubulin heterodimers, resulting in increased primary and secondary dendrite numbers (Akum et al., 2004). We have found that cypin is a core regulator of dendritogenesis, and two well-studied regulators of dendrite number, brain-derived neurotrophic factor (BDNF) and the small GTPase RhoA, act via cypin-dependent pathways (Chen and Firestein, 2007; Kwon et al., 2011). Recently, we developed new Sholl analyses to determine how BDNF acts at subregions of the arbor and found novel action of BDNF at terminal regions of the arbor (Langhammer et al., 2010). Since cypin promotes local microtubule assembly (Akum et al., 2004), and our previous studies have only assessed the effects of overexpression and knockdown of cypin by either counting primary and secondary dendrites (Akum et al., 2004; Charych et al., 2006; Fernandez et al., 2008) or by using conventional Sholl analysis (Chen and Firestein, 2007; Kwon et al., 2011), it is not yet known whether cypin has region-specific effects on the dendritic arbor. Here, we alter cypin protein levels in cultured rat hippocampal neurons by overexpression from day *in vitro* (DIV) 6–10 and from DIV 10–12, and we apply several types of Sholl analyses on specific regions of the dendritic arbor. Our data show that cypin promotes proximal branching at both time points and that these increases are order-specific. These results suggest that that traditional Sholl analysis and dendrite number counts are not sufficient to describe cypin-promoted changes to the dendritic arbor.

## Materials and methods

### Primary culture and transfection of hippocampal neurons

This study was carried out in accordance with the recommendations of the National Institute of Health's Guide for the Care and Use of Laboratory Animals (DHHS Publication No. [NIH] 85-23 and all subsequent revisions thereof) and to the Public Health Service Policy on Humane Care and Use of Laboratory Animals followed by Rutgers Institutional Animal Care and Use Committee. The protocol was approved by the Rutgers Institutional Animal Care and Use Committee.

Hippocampal neurons were isolated from embryonic rats at day 18 of gestation (E18) as we have previously described (Firestein et al., 1999). After isolation, the hippocampi were dissociated via manual trituration and plated at a density of 2 × 10^5^/well on 12-mm glass coverslips (Fisher) in 24-well plates (Corning). Coverslips were coated with 0.5 mg/mL poly-D-lysine (PDL; Sigma) for at least 1 h at 37°C prior to plating cells. Cultures were maintained in Neurobasal medium supplemented with B27, GlutaMAX, and penicillin/streptomycin (all from Life Technologies) in a humidified 37°C incubator with 5% CO_2_. Cells were grown for 6 days *in vitro* (DIV) or 10 DIV prior to transfection.

A subset of neurons were transfected at DIV 6 with pEGFP-C1 or pEGFP-C1-cypin and pmRFP using Lipofectamine LTX and Plus reagent according to the manufacturer's instructions (Kwon et al., 2011) and fixed with 4% paraformaldehyde in phosphate-buffered saline (PBS), pH 7.4, at DIV 10. Additional neurons were transfected at DIV 10 with pEGFP-C1 or pEGFP-C1-cypin using Lipofectamine 2000 according to the manufacturer's instructions and fixed at DIV 12 for imaging and analysis.

### Immunostaining and imaging

At the appropriate DIV, neurons were fixed with 4% paraformaldehyde in PBS for 15 min and incubated in blocking buffer (PBS containing 0.1% Triton X-100, 2% normal goat serum, and 0.02% sodium azide) for 1 h. All antibodies were diluted in blocking buffer. Cells were incubated with primary antibody for 2 h at room temperature. Primary antibodies were used at a concentration of 1:500 and included mouse anti-MAP2 (BD Pharmigen), chicken anti-GFP (Rockland), and rabbit anti-cypin (Chen and Firestein, 2007). After primary antibody incubation, coverslips were washed three times with PBS and incubated with secondary antibody for 1 h at room temperature. Secondary antibodies were used at a concentration of 1:250 and included Alexa Fluor 488 donkey anti-chicken, Alexa Fluor 555 donkey anti-rabbit, and Alexa Fluor 647 donkey anti-mouse (all from Life Technologies). After secondary antibody incubation, coverslips were washed twice with PBS and incubated with Hoechst dye for 5 min at room temperature to stain nuclei. Coverslips were washed one final time with PBS and mounted onto glass microscope slides with Fluoromount G (Southern Biotechnology). Transfected cells were visualized by immunofluorescence on an Olympus Optical IX 50 microscope (Tokyo, Japan) with a Cooke SensiCam charge-coupled device (CCD) cooled camera fluorescence imaging system and Image Pro software (Media Cybernetics).

### Assessment of dendrite number using semi-automated sholl analysis and statistics

Semi-automated Sholl analysis was used as previously described (Kutzing et al., 2010; Langhammer et al., 2010). Briefly, 8-bit images of hippocampal neurons were traced using the NeuronJ plugin (Meijering et al., 2004) for ImageJ (NIH, Bethesda, MD), and tracing files (*.ndf files) were generated. The data were organized and converted to SWC files (Cannon et al., 1998) using MATLAB (Mathworks), and the connectivity of the tracings was checked in NeuronStudio (Rodriguez et al., 2006). Once the tracings were finalized in NeuronStudio, the data were exported to Excel using MATLAB. Prism (Graphpad) was used for all statistical analyses. For analysis of Sholl curves, two-way ANOVA was used followed by Bonferroni Multiple Comparisons test. For analysis of dendrite numbers, Student *t*-tests were used, and Welch's correction was included when appropriate. All tracings and analyses were performed with the experimenter blinded to the condition. A subset of neurons for this study were retraced from images analyzed in Kwon et al. (2011). All analyses of the retraced images presented here are new, and data generated from non-conventional Sholl analyses (RIT and Tips-In) are novel and were not included in Kwon et al. (2011). Neurons were counted only when at least two authors agreed that they were viable without access to condition information. All dendrites are defined as not branching or resulting in bifurcation (Van Pelt and Verwer, 1985, 1986; Verwer and Van Pelt, 1990).

### Labeling schemes used for analysis

**Figure 1:**
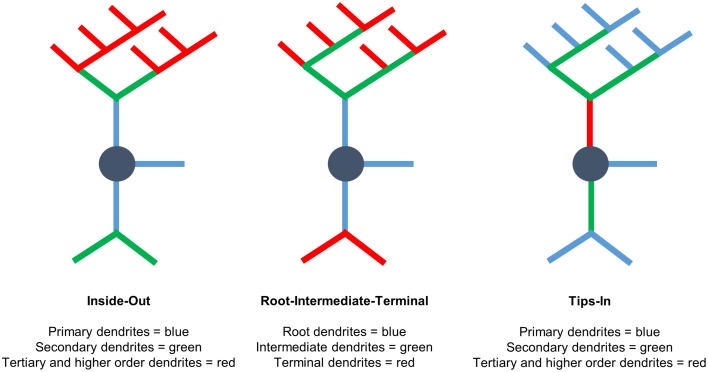
**Three different Sholl analyses used to assess the effects of cypin overexpression on the dendritic arbor**. **(Left)** Inside-Out Sholl analysis is conventional Sholl analysis (Langhammer et al., 2010). Dendrites that extend from the cell body are defined as primary dendrites, those that emanate from primary dendrites are secondary dendrites, those that emanate from secondary dendrites are tertiary dendrites, and so on. Dendrites classified as tertiary and higher are grouped together. **(Middle)** In the Root-Intermediate-Terminal (RIT) scheme (Langhammer et al., 2010), root dendrites emerge from the cell body, and terminal dendrites are those dendrites that do not branch further. All other dendrites are considered intermediate dendrites. **(Right)** Tips-In analysis (Rodriguez, 2007) defines terminal dendrites as primary dendrites. Secondary dendrites are dendrites that are one order in from the outermost dendrite and can be the penultimate dendrite or a root dendrite with only one branch, as shown in the figure. Tertiary and higher order dendrites are one order closer to the soma after the penultimate (secondary) dendrite. These dendrites correspond to primary dendrites in the Inside-Out labeling scheme that branch at least twice. This is a novel analysis, presented within this paper.

We use three labeling schemes to analyze cypin-promoted changes to the dendritic arbor (Figure 1). It is important to note that Sholl data are always reported at distance from the cell body in μm, regardless of the labeling scheme. Inside-Out Sholl analysis is conventional Sholl analysis (Langhammer et al., 2010). Dendrites that extend from the cell body are defined as primary dendrites, and those that emanate from primary dendrites are secondary dendrites. Dendrites that emanate from secondary dendrites are tertiary dendrites, and so on. In this labeling scheme, dendrites classified as tertiary and higher are grouped together. In the Root-Intermediate-Terminal (RIT) scheme (Langhammer et al., 2010), root dendrites emerge from the cell body, and terminal dendrites are those dendrites that do not branch further. All other dendrites are labeled as intermediate dendrites. Tips-In analysis (Rodriguez, 2007) is the opposite of Inside-Out analysis. This scheme defines the outermost, terminal dendrites as primary dendrites. Secondary dendrites are dendrites that are one order in from the outermost dendrite; they can be the penultimate dendrite or a root dendrite with only one branch, as shown in the right panel of Figure 1. Tertiary and higher order dendrites are one order closer to the soma after the penultimate (secondary) dendrite. These dendrites correspond to primary dendrites in the Inside-Out labeling scheme that branch at least twice.

## Results

### Sholl analysis for neurons overexpressing cypin from DIV 6–10

**Figure 2:**
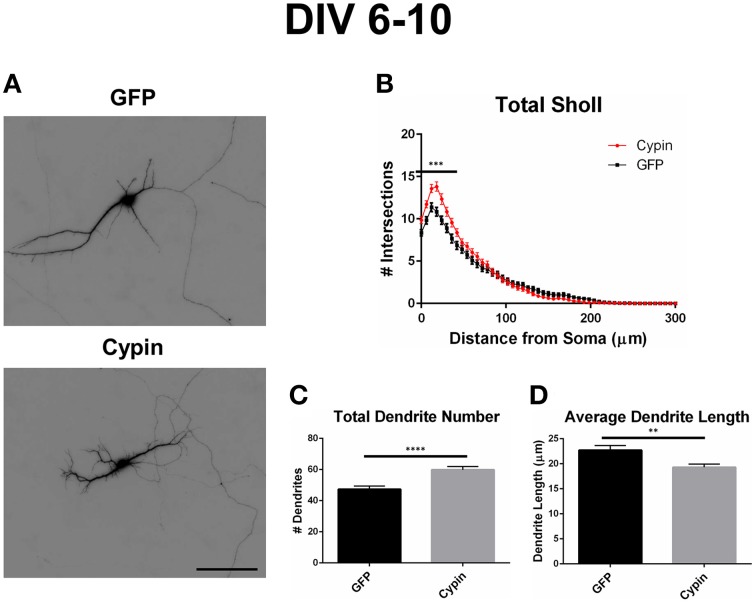
**Overexpression of cypin from DIV 6–10 increases proximal branching and total dendrite number but decreases average dendrite length**. **(A)** Representative images of hippocampal neurons overexpressing GFP or GFP-cypin (cypin) from DIV 6–10. Scale bar = 100 μm. **(B)** Sholl analysis of all orders of branches (Total Sholl) shows that overexpression of cypin significantly increases dendrite branching at 0–42 μm from the cell body (^***^p < 0.001). Statistics were calculated using Two-Way ANOVA followed by Bonferroni multiple comparisons test. **(C)** Overexpression of cypin results in a significant increase in the total number of dendrites (^****^p < 0.0001). Total number of dendrites represents sum of all dendrites, regardless to what category they belong. Statistics were calculated by unpaired, two-tailed Student's *t*-test. **(D)** Overexpression of cypin results in a significant decrease in the average length of dendrites (^**^p < 0.001). Average length is the mean length of all dendrites, regardless to what category they belong. Statistics were calculated by unpaired, two-tailed Student's *t*-test with Welch's correction. Error bars indicate SEM. *n* = 50 neurons for GFP, and *n* = 55 neurons for cypin.

As illustrated in the representative images in Figure 2A, overexpression of cypin from DIV 6–10 promotes dendrite branching in hippocampal neurons, in agreement with our previous work (Akum et al., 2004; Chen and Firestein, 2007; Fernandez et al., 2008; Kwon et al., 2011). Sholl analysis performed with all branch orders grouped (Total Sholl, Figure 2B), as is standard in the field, is the same analysis regardless of whether we perform Inside-Out, Root-Intermediate-Terminal (RIT), or Tips-In analysis. Total Sholl analysis shows that cypin significantly increases proximal branches at 0–42 μm from the soma when overexpressed from DIV 6–10. The causes for this change can be parsed out when examining Sholl curves for different branch categories and comparing these differences among the three methods of analysis.

**Figure 3:**
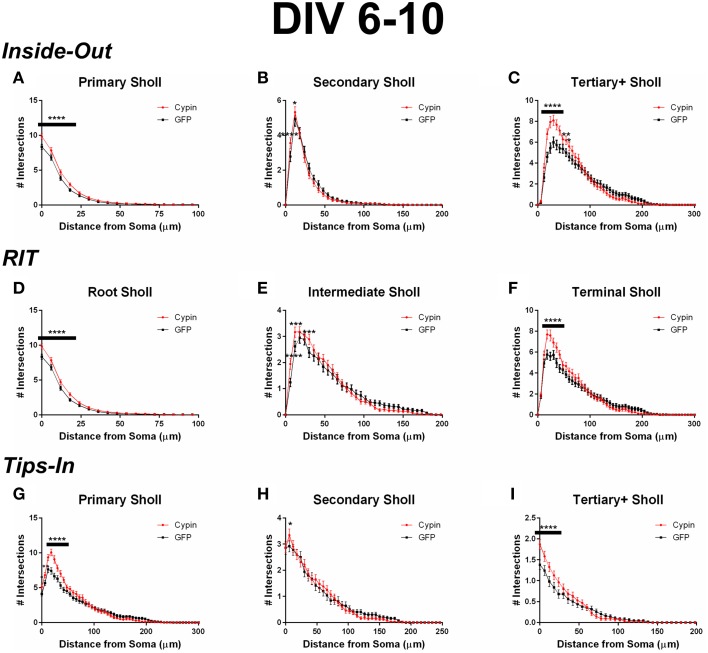
**Sholl analysis using three different labeling methods for neurons overexpressing cypin from DIV 6–10**. **(A–C)** Sholl analysis using Inside-Out (conventional) labeling method. **(A)** Sholl analysis of primary dendrites (Primary Sholl) shows that overexpression of cypin significantly increases branching at 0–18 μm (^****^p < 0.0001). **(B)** Sholl analysis of secondary dendrites (Secondary Sholl) shows that overexpression of cypin significantly increases dendrites at 6 and 12 μm from the soma (^****^p < 0.0001 and ^*^p < 0.05, respectively). **(C)** Sholl analysis of tertiary and higher order dendrites (Tertiary+ Sholl) shows that overexpression of cypin significantly increases dendrites at 18–42 μm from the soma (^****^p < 0.0001) and at 54 μm (^**^p < 0.01) and 60 μm (^*^p < 0.05) from the soma. **(D–F)** Sholl analysis using RIT labeling method. (**D**) Sholl analysis of root dendrites (Root Sholl) shows that overexpression of cypin significantly increases branching at 0–18 μm from the cell body (^****^p < 0.0001). **(E)** Sholl analysis of intermediate dendrites (Intermediate Sholl) shows that overexpression of cypin significantly increases dendrites at 6 μm (^****^p < 0.0001) from the soma and at 12 and 30 μm from the soma (both ^***^p < 0.001). **(F)** Sholl analysis of terminal dendrites (Terminal Sholl) shows that overexpression of cypin significantly increases dendrites at 18–42 μm from the soma (^****^p < 0.0001). **(G–I)** Sholl analysis using Tips-In labeling method. **(G)** Sholl analysis of primary dendrites shows that overexpression of cypin significantly increases branching at 0 μm (^*^p < 0.05), 6 μm (^**^p < 0.01), and 12–42 μm from the soma (^****^p < 0.0001). **(H)** Sholl analysis of secondary dendrites shows that overexpression of cypin significantly increases branching at 6 μm from the soma (^*^p < 0.05). **(I)** Sholl analysis of tertiary and higher order dendrites shows that overexpression of cypin significantly increases dendrite branching at 0–24 μm from the soma (^****^p < 0.0001). Statistics were calculated using Two-Way ANOVA followed by Bonferroni multiple comparisons test. Error bars indicate SEM. *n* = 50 neurons for GFP, and *n* = 55 neurons for cypin.

When analyzing the dendritic arbor using the Inside-Out method, cypin overexpression promotes the greatest increase in primary branches, branches that emerge from the soma, and higher order branches (tertiary and above). Primary branches significantly increase at 0–18 μm from the soma when cypin is overexpressed (Figure 3A). Secondary branches, which emerge from primary dendrites, significantly increase at 6–12 μm from the soma (Figure 3B). Higher order dendrites significantly increase at 18–42 μm and at 54–60 μm from the soma in neurons overexpressing cypin (Figure 3C). These results indicate that, at DIV 6–10, when dendritic branches extend from primary and secondary branches and very little pruning has yet to occur (i.e., stage 4) (Dotti et al., 1988; Akum et al., 2004), cypin exerts the greatest effects on primary and higher order branches (tertiary and above) but not on secondary branches.

When analyzing the dendritic arbor using the RIT method, cypin overexpression significantly changes all categories of dendrites. Like the Inside-Out method, significant changes occur in proximal dendrites less than 100 μm from the soma. Because primary dendrites in the Inside-Out method and root dendrites in the RIT method are defined as the same, changes observed in root dendrites are the changes observed in primary dendrites: cypin overexpression significantly increases root dendrites at 0–42 μm from the soma (Figure 3D). This method of Sholl analysis shows a difference from that of Inside-Out Sholl analysis when comparing secondary dendrites and intermediate dendrites (Figure 3E). Intermediate dendrites include all dendrites that are not root dendrites or terminal dendrites. Significant increases in dendrites of neurons overexpressing cypin are observed at 6–12 μm from the soma, similar to changes seen in secondary dendrites analyzed by the Inside-Out method. Additionally, an increase in intermediate dendrites at 30 μm from the soma is also observed. These results indicate that the significant increases observed at 6 and 12 μm are due to increased secondary dendrites but that the increase at 30 μm is due to increased higher order, intermediate branches. The Sholl curves for terminal dendrites show similar increases to those seen in tertiary and higher order dendrites: neurons overexpressing cypin have significantly increased dendrite branching at 18–42 μm from the soma (Figure 3F). Interestingly, the increases observed at 54 and 60 μm from the soma that are observed for tertiary and higher order dendrites are not observed for terminal dendrites, indicating that terminal dendrites closer to the cell body (within 50 μm) are most affected by cypin overexpression.

When analyzing the dendritic arbor using the Tips-In method, cypin overexpression from DIV 6–10 exerts the greatest effect on primary dendrites and on tertiary and higher order dendrites. In this labeling scheme, primary dendrites are the outermost (terminal) dendrites. The resulting curve from this analysis is distinct from the terminal Sholl curve resulting from the RIT method, likely because primary dendrites in this case are a combination of terminal dendrites as well as root dendrites that do not branch (see Figure 1, right panel). For primary dendrites in the Tips-In scheme, significant increases are observed at 0–42 μm from the soma (Figure 3G), which are the same distances observed for increased dendrites resulting from Total Sholl analysis (Figure 2B). Secondary dendrites in this scheme are dendrites that are one order in from the outermost dendrite, either the penultimate dendrite or a root dendrite with only one branch (Figure 1, right panel). This curve is distinct from the intermediate Sholl curve from the RIT method and the secondary Sholl curve from the Inside-Out method. Significant increases are only observed at 6 μm from the soma (Figure 3H), indicating that the penultimate dendrite is only affected by cypin overexpression at distances very close to the soma. Finally, dendrites labeled as tertiary and higher order are one order closer to the soma after the penultimate (secondary) dendrite; they are the antepenultimate (third to last) dendrite. These dendrites correspond to primary dendrites in the Inside-Out labeling scheme that branch at least twice (Figure 1, right panel). For tertiary and higher order dendrites, significant increases are observed at 0–24 μm away from the soma (Figure 3I).

### Dendrite number for neurons overexpressing cypin from DIV 6–10

Cypin overexpression from DIV 6–10 affects the total number of dendrites and dendrites of specific orders, depending on the labeling scheme. For all labeling schemes, the total dendrite count is identical by definition (*p* > 0.9999 as determined by One-Way ANOVA), and cypin overexpression from DIV 6–10 significantly increases dendrites (Figure 2C). The different labeling schemes indicate unique regulation of dendrite number by cypin.

**Figure 4:**
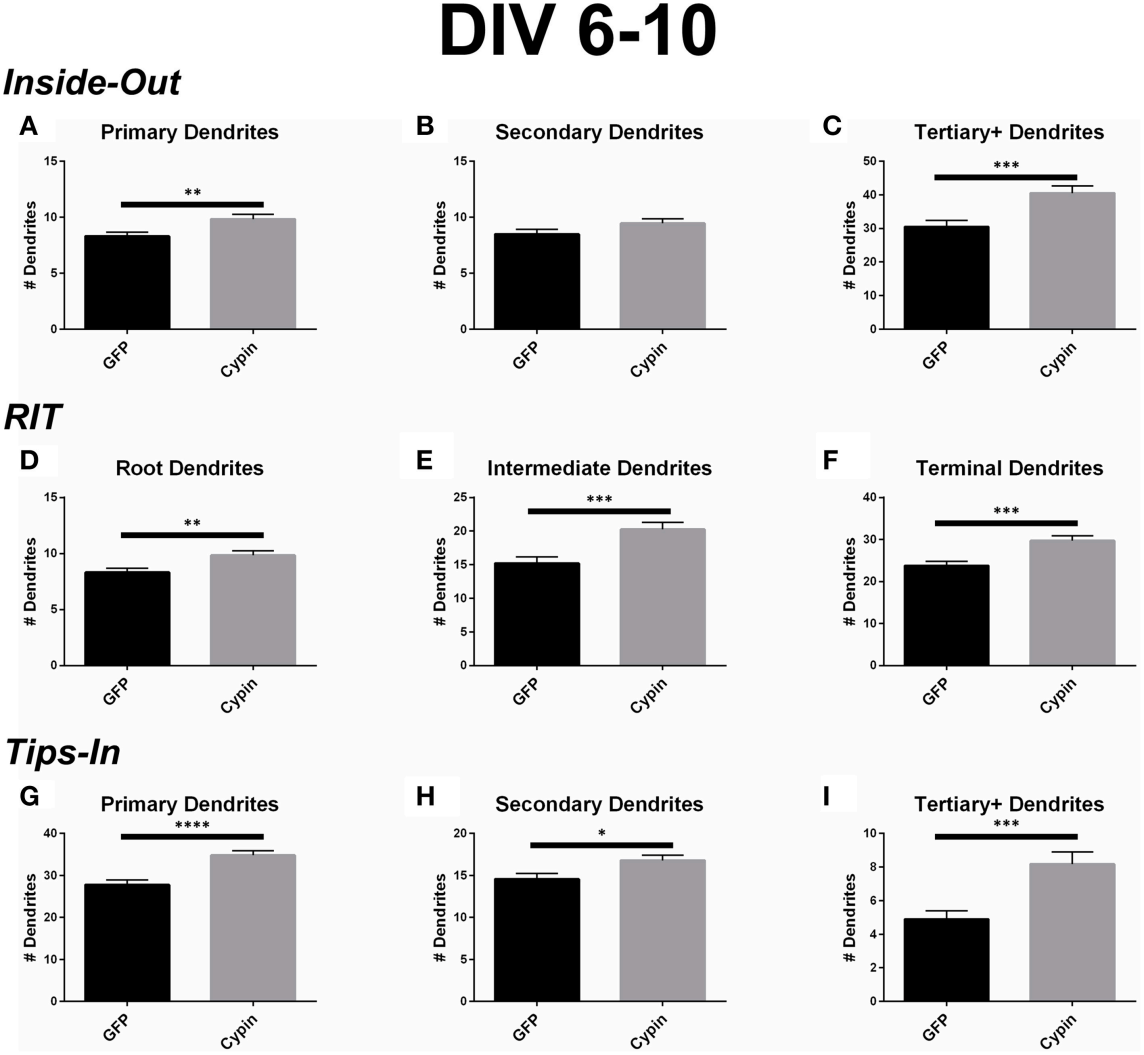
**Cypin increases the number of dendrites by specifically targeting certain categories of dendrites when overexpressed in neurons from DIV 6–10**. **(A–C)** Dendrite numbers divided into categories using Inside-Out (conventional) labeling method. **(A)** Overexpression of cypin significantly increases the number of primary dendrites (^**^p < 0.01). **(B)** Overexpression of cypin does not significantly increase the number of secondary dendrites. **(C)** Overexpression of cypin significantly increases the number of tertiary and higher order dendrites (^***^p < 0.001). **(D–F)** Dendrite numbers divided into categories using RIT labeling method. **(D)** Overexpression of cypin significantly increases the number of root dendrites (^**^p < 0.01). **(E)** Overexpression of cypin significantly increases the number of intermediate dendrites (^***^p < 0.001). **(F)** Overexpression of cypin significantly increases the number of terminal dendrites (^***^p < 0.001). **(G–I)** Dendrite numbers divided into categories using Tips-In labeling method. **(G)** Overexpression of cypin significantly increases the number of primary dendrites (^****^p < 0.0001). **(H)** Overexpression of cypin significantly increases the number of secondary dendrites (^*^p < 0.05). **(I)** Overexpression of cypin significantly increases the number of tertiary and higher order dendrites (^***^p < 0.001). Statistics calculated by unpaired, two-tailed Student's *t*-test. Error bars indicate SEM. *n* = 50 neurons for GFP, and *n* = 55 neurons for cypin.

For the Inside-Out labeling scheme, cypin overexpression significantly increases primary dendrites (Figure 4A) as well as tertiary and higher order dendrites (Figure 4C), with no significant effect on secondary dendrites (Figure 4B). This labeling scheme points to cypin-promoted increases in primary and higher order (>secondary) dendrite number.

For the RIT labeling scheme, overexpression of cypin significantly increases all dendrite types; root, intermediate, and terminal dendrite numbers are significantly increased (Figures 4D–F). When comparing these graphs to the Inside-Out graphs, it becomes clear that different labeling schemes demonstrate cypin-promoted effects on dendrite number differently. As with the Sholl curves, the difference in root dendrites is identical for that of primary dendrites because the two types of dendrites are identical (Figure 4D). While there is no significant difference observed for secondary dendrites for the Inside-Out method, there is a significant increase in intermediate dendrites when cypin is overexpressed (Figure 4E). This is also reflected in the Sholl curves, with an additional significant difference at 30 μm from the soma for intermediate dendrites (Figure 3E) when compared to secondary dendrites (Figure 3B). Additionally, terminal dendrites significantly increase due to cypin overexpression, indicating that increased branching is due to increased intermediate branches (Figure 4F).

Interestingly, unlike the Inside-Out method, the Tips-In method shows significantly increased numbers of dendrites for all categories (Figures 4G–I). As with Sholl analysis for this method, the primary dendrites are terminal dendrites or root dendrites that do not branch, and these types of dendrites are significantly increased for neurons overexpressing cypin from DIV 6–10 (Figure 4G). Secondary dendrites, defined under this labeling scheme, are either the penultimate dendrite or a root dendrite with only one branch. In this case, they significantly increase with cypin overexpression (Figure 4H). Finally, tertiary and higher order dendrites are primary dendrites with two or more branches. We also observe a significant increase in these branches as a result of cypin overexpression (Figure 4I).

### Dendrite length for neurons overexpressing cypin from DIV 6–10

In addition to the effects of cypin overexpression from DIV 6–10 on dendrite number and spatial arrangement, cypin overexpression affects the lengths of dendrites. As shown in Figure 2C, overexpression of cypin significantly increases the total number dendrites, but as shown in Figure 2D, it also significantly decreases the average dendritic length, perhaps due to the exhaustion of a limiting reagent required for dendrite growth (Charych et al., 2006).

**Figure 5:**
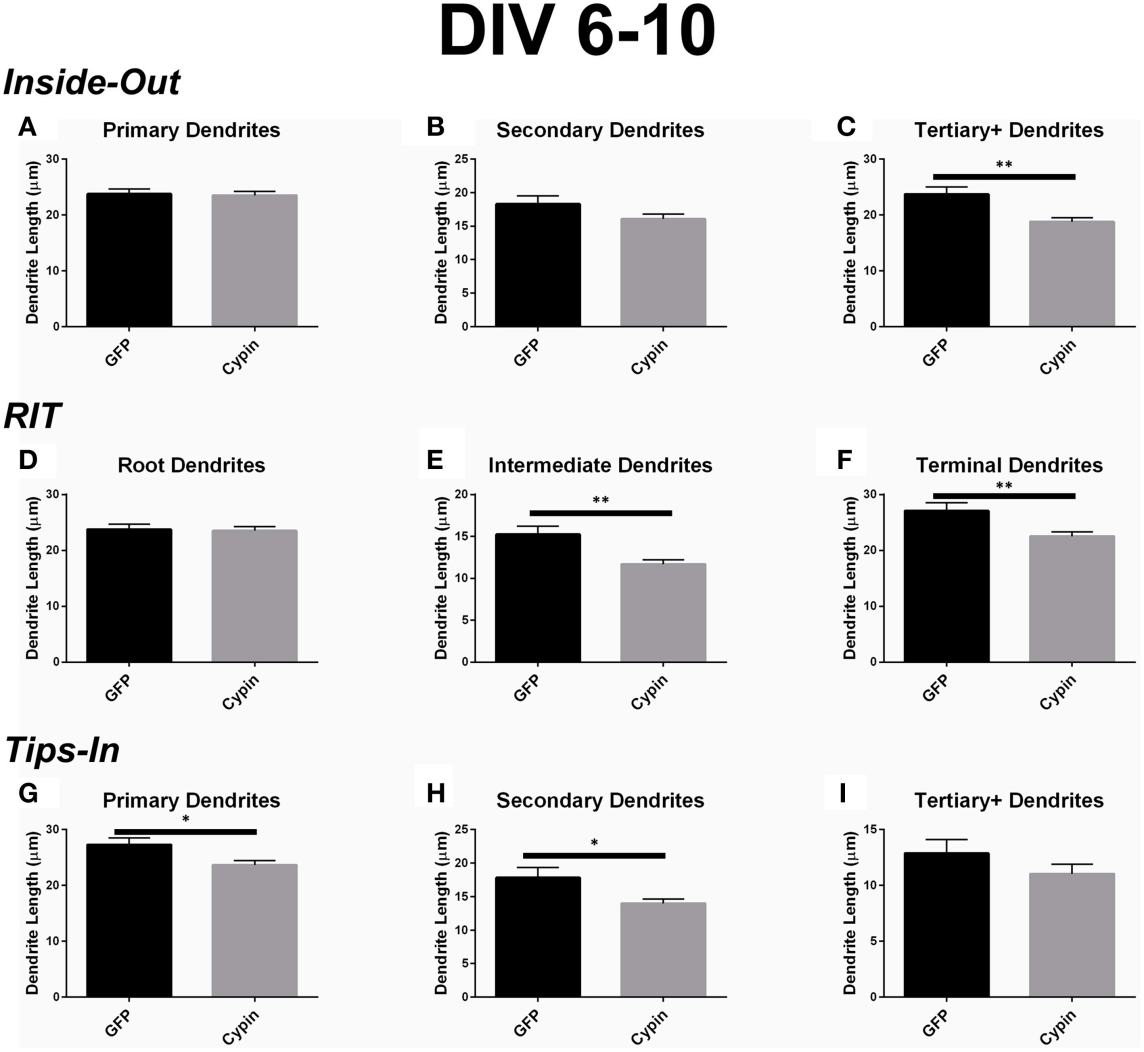
**Cypin decreases the length of dendrites by specifically targeting certain categories of dendrites when overexpressed in neurons from DIV 6-10. (A–C)** Average dendrite length divided into categories using Inside-Out (conventional) labeling method. **(A)** Cypin overexpression does not significantly change the length of primary dendrites. **(B)** Cypin overexpression does not significantly decrease the length of secondary dendrites. **(C)** Cypin overexpression significantly decreases the length of tertiary and higher order dendrites (^**^p < 0.001). For **(A,B)** statistics were calculated by unpaired, two-tailed Student's *t*-tests, and for **(C)**, statistics were calculated by unpaired, two-tailed Student's *t*-tests with Welch's correction. **(D–F)** Average dendrite length divided into categories using RIT labeling method. **(D)** Cypin overexpression does not significantly change the length of root dendrites. **(E)** Cypin overexpression significantly decreases the length of intermediate dendrites (^**^p < 0.001). **(F)** Cypin overexpression significantly decreases the length of terminal dendrites (^**^p < 0.001). For **(D)**, statistics were calculated by unpaired, two-tailed Student's *t*-tests, and for **(E,F)**, statistics were calculated by unpaired, two-tailed Student's *t*-tests with Welch's correction. **(G–I)** Average dendrite length divided into categories using Tips-In labeling method. **(G)** Cypin overexpression significantly decreases the length of primary dendrites (^*^p < 0.05). **(H)** Cypin overexpression significantly decreases the length of secondary dendrites (^*^p < 0.05). **(I)** Cypin overexpression does not significantly decrease the length of tertiary and higher order dendrites. For **(G–I)**, statistics were calculated by unpaired, two-tailed Student's *t*-tests with Welch's correction. Error bars indicate SEM. *n* = 50 neurons for GFP, and *n* = 55 neurons for cypin.

When analyzed according to the Inside-Out labeling method, overexpression of cypin significantly decreases the length of tertiary and higher order dendrites (Figure 5C). Interestingly, the length of primary dendrites is not affected (Figure 5A) although their numbers are increased (as shown in Figure 4A). Secondary dendrite length is not affected by cypin overexpression (Figure 5B). These data indicate that overexpression of cypin specifically changes the length of higher order dendrites.

The dendrite length data from the RIT method complement the data gathered using the Inside-Out method. While cypin overexpression does not affect the length of root dendrites (Figure 5D), it significantly decreases the lengths of intermediate and terminal dendrites (Figure 5E and Figure 5F, respectively). Only tertiary and higher order dendrite numbers significantly decrease with cypin overexpression when analyzed using the Inside-Out method, indicating that analysis of two different classes (intermediate and terminal) are combined, thus eliminating information. Additionally, while no significant difference in length is observed for secondary dendrites (Figure 5B), a significant decrease in intermediate dendrite length results from cypin overexpression (Figure 5E). Based on these data, cypin overexpression affects the length of intermediate branches, most likely higher order (>secondary) dendrite branches, and terminal branches.

Finally, the data for the Tips-In method indicate that overexpression of cypin significantly decreases the length of primary and secondary dendrites (Figures 5G,H). Primary dendrites are terminal dendrites or root dendrites that have not branched, and secondary dendrites are the penultimate branch or a root dendrite that has branched once (Figure 1, right panel). While these are two very different categories of branches, cypin overexpression significantly decreases the lengths of both of these types of branches. Cypin overexpression does not result in a change in length of tertiary and higher order dendrites (Figure 5I).

### Sholl analysis for neurons overexpressing cypin from DIV 10–12

**Figure 6:**
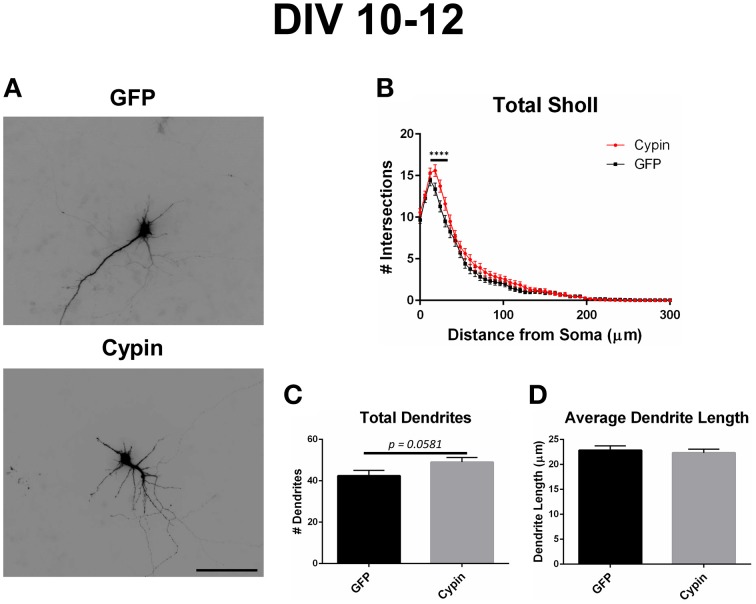
**Cypin overexpression from DIV 10–12 increases proximal branching with no significant effect on total dendrite number or average dendrite length**. **(A)** Representative images of hippocampal neurons overexpressing GFP or GFP-cypin (cypin) from DIV 10–12. Scale bar = 100 μm. **(B)** Sholl analysis for all orders of branches (Total Sholl) shows that overexpression of cypin significantly increases dendrite branching at 18–30 μm from the soma (^****^p < 0.0001). Statistics were calculated using Two-Way ANOVA followed by Bonferroni multiple comparisons test. **(C)** Cypin overexpression results in an increase that approaches significance in the number of total dendrites (*p* = 0.0581). Total number of dendrites represents sum of all dendrites, regardless to what category they belong. **(D)** Cypin overexpression does not significantly change average dendrite length. Average length is the mean length of all dendrites, regardless to what category they belong. For **(C,D)**, statistics were calculated by unpaired, two-tailed Student's *t*-test. Error bars indicate SEM. *n* = 35 neurons for GFP, and *n* = 51 neurons for cypin.

As we previously reported (Akum et al., 2004; Chen and Firestein, 2007; Fernandez et al., 2008; Kwon et al., 2011), overexpression of cypin from DIV 10–12 also promotes dendrite branching in hippocampal neurons as demonstrated in the representative images in Figure 6A. Sholl analysis performed with all branch orders grouped (Total Sholl, Figure 6B) is the same analysis regardless of which labeling method is used. Total Sholl analysis shows that overexpression of cypin significantly increases proximal branches at 18–30 μm from the soma when it is overexpressed from DIV 10–12. As with our analysis of neurons overexpressing cypin from DIV 6–10, we can identify the mechanism by which this change occurs by examining Sholl curves for different branch categories and comparing these differences among the three analysis methods.

**Figure 7:**
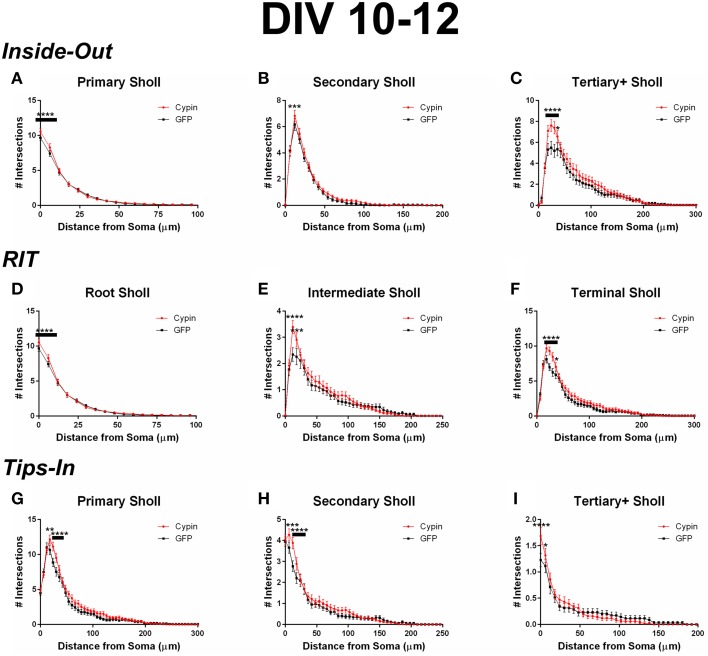
**Sholl analysis using three different labeling methods for neurons overexpressing cypin from DIV 10–12**. **(A–C)** Sholl analysis using Inside-Out (conventional) labeling method. **(A)** Sholl analysis of primary dendrites (Primary Sholl) shows that overexpression of cypin significantly increases branching at 0–6 μm from the soma (^****^p < 0.0001). **(B)** Sholl analysis of secondary dendrites (Secondary Sholl) shows that overexpression of cypin significantly increases branching at 12 μm from the soma (^***^p < 0.01). **(C)** Sholl analysis of tertiary and higher order dendrites (Tertiary + Sholl) shows that overexpression of cypin significantly increases branching at 18–30 μm from the soma (^****^p < 0.0001) and at 36 μm from the soma (^*^p < 0.05). **(D–F)** Sholl analysis using RIT labeling method. **(D)** Sholl analysis of root dendrites (Root Sholl) shows that overexpression of cypin significantly increases branching at 0–6 μm from the soma (^****^p < 0.0001). **(E)** Sholl analysis of intermediate dendrites (Intermediate Sholl) shows that overexpression of cypin significantly increases dendrite branching at 12 μm (^****^p < 0.0001) and at 18 μm (^***^p < 0.001) from the soma. **(F)** Sholl analysis of terminal dendrites (Terminal Sholl) shows that overexpression of cypin significantly increases dendrite branching at 18–30 μm from the soma (^****^p < 0.0001) and at 36 μm from the soma (^*^p < 0.05). **(G–I)** Sholl analysis using Tips-In labeling method. **(G)** Sholl analysis of primary dendrites shows that overexpression of cypin increases branching at 18 μm from the soma (^**^p < 0.01) and at 24–30 μm from the soma (^****^p < 0.0001). **(H)** Sholl analysis of secondary dendrites shows that overexpression of cypin increases dendrite branching at 6 μm from the soma (^***^p < 0.001) and at 12–18 μm from the soma (^****^p < 0.0001). **(I)** Sholl analysis of tertiary and higher order dendrites shows that overexpression of cypin significantly increases branching at the soma (0 μm, ^****^p < 0.0001) and at 6 μm from the soma (^*^p < 0.05). Statistics were calculated using Two-Way ANOVA followed by Bonferroni multiple comparisons test. Error bars indicate SEM. *n* = 35 neurons for GFP, and *n* = 51 neurons for cypin.

When analyzing the dendritic arbor using the Inside-Out method, cypin overexpression from DIV 10–12 promotes proximal branching in all branch types. Primary branches significantly increase at 0–6 μm from the soma when cypin is overexpressed (Figure 7A). Secondary dendrites, which emerge from primary dendrites, significantly increase at 12 μm from the soma (Figure 7B). Higher order dendrites significantly increase at 18–36 μm from the soma in neurons overexpressing cypin (Figure 7C). These results indicate that during DIV 10–12, at the end of the active dendrite branching period in our cultured neurons (i.e., stage 4) (Dotti et al., 1988; Akum et al., 2004), cypin overexpression promotes increases in all branch types at specific distances from the soma.

Cypin overexpression also promotes increases in all categories of dendrites when analyzed by the RIT labeling method. As with the Inside-Out method, significant changes occur in proximal dendrites less than 50 μm from the soma. Primary dendrites identified by the Inside-Out method and root dendrites identified by the RIT method are identical, and thus, the changes observed in these dendrite categories are the same: a significant increase in root dendrites at 0–6 μm from the soma (Figure 7D). There is a difference, however, when comparing secondary dendrites from the Inside-Out method and intermediate dendrites from the RIT method because intermediate dendrites include all dendrites that are not root or terminal dendrites. For neurons overexpressing cypin, significant increases in intermediate dendrites are observed at 12–18 μm from the soma using the RIT method (Figure 7E), whereas there is a sole significant increase at 12 μm from the soma for secondary dendrites when using the Inside-Out method (Figure 7B). This difference indicates that the significant increase at 12 μm is due to increased secondary dendrites, whereas the increase at 18 μm is due to increases in higher order dendrites. The Sholl curves for terminal dendrites analyzed using the RIT method show similar increases to those identified as tertiary and higher order using the Inside-Out method. Neurons overexpressing cypin show significantly increased branching 18–36 μm from the soma (Figure 7F). These results reveal that cypin-promoted increases in higher order dendrites at these distances are specifically due to increased terminal dendrite branching.

When analyzing the dendritic arbor using the Tips-In method, cypin overexpression results in increased dendrites of all categories. Primary branches are the outermost, or terminal, dendrites in this labeling scheme. The Sholl curve for these branches is similar to that of the terminal branches for RIT but has some important differences. For the Tips-In labeling scheme, primary dendrites include terminal dendrites and root dendrites that do not branch (Figure 1, right panel). Primary dendrites significantly increase in neurons overexpressing cypin at 18–30 μm from the soma (Figure 7G), which are the same distances observed for increased dendrites identified by Total Sholl analysis (Figure 6B). These data indicate that the outermost dendrites and root dendrites that do not branch are responsible for the overall increases observed. Secondary dendrites in this scheme are dendrites that are one order in from the outermost dendrite, either the penultimate dendrite or a root dendrite with only one branch (Figure 1, right panel). The Sholl curve resulting from analysis of secondary dendrites from the Tips-In method is distinct from the intermediate Sholl curve from the RIT method and the secondary Sholl curve from the Inside-Out method. For the Tips-In method, significant increases in branching occur at 6–18 μm from the soma for neurons overexpressing cypin (Figure 7H). In contrast, intermediate dendrites identified by the RIT method significantly increase at 12–18 μm from the soma (Figure 7E). These differences can be explained by how dendrites are grouped. Secondary dendrites, as defined by the Tips-In scheme, include intermediate dendrites and root dendrites with only one branch. Thus, the additional significant increase at 6 μm could be due to an increase in root dendrites with only one branch. Finally, dendrites labeled as tertiary and higher order are the antepenultimate (third to last) dendrite. These dendrites correspond to root dendrites that branch twice or more. For tertiary and higher order dendrites of the Tips-In scheme, significant increases occur at 0–6 μm from the soma (Figure 7I). These increases correspond to those observed for primary dendrites identified by the Inside-Out scheme and root dendrites identified by the RIT scheme.

### Dendrite number for neurons overexpressing cypin from DIV 10–12

**Figure 8:**
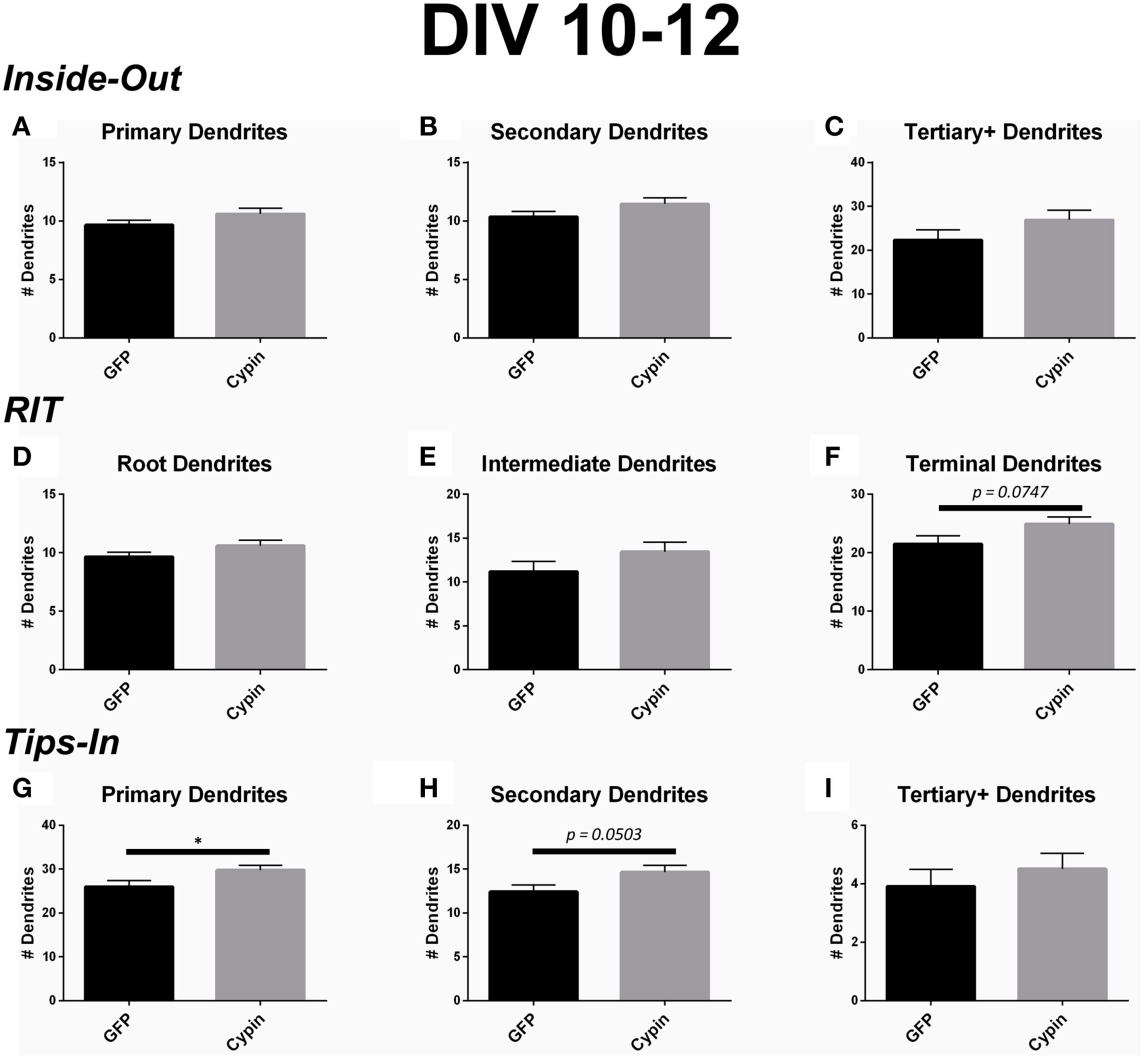
**Cypin increases outermost dendrites when overexpressed in neurons from DIV 10–12**. (**A–C)** Dendrite numbers divided into categories using Inside-Out (conventional) labeling method. **(A)** Cypin overexpression does not increase the number of primary dendrites. **(B)** Cypin overexpression does not increase the number of secondary dendrites. **(C)** Cypin overexpression does not increase the number of tertiary and higher order dendrites. For **(A,B)**, statistics were calculated by unpaired, two-tailed Student's *t*-tests with Welch's correction, and for **(C)**, statistics were calculated by unpaired, two-tailed Student's *t*-tests. **(D–F)** Dendrite numbers divided into categories using RIT labeling method. **(D)** Cypin overexpression does not increase the number of root dendrites. **(E)** Cypin does not increase the number of intermediate dendrites. **(F)** Cypin overexpression causes an increase in the number of terminal dendrites that approaches significance (*p* = 0.0747). For **(D,E)**, statistics were calculated by unpaired, two-tailed Student's *t*-tests with Welch's correction, and for **(F)**, statistics were calculated by unpaired, two-tailed Student's *t*-tests. **(G–I)** Dendrite numbers divided into categories using Tips-In labeling method. **(G)** Cypin overexpression significantly increases the number of primary dendrites (^*^p < 0.05). **(H)** Cypin overexpression results in an increase that approaches significance in the number of secondary dendrites (*p* = 0.0503). **(I)** Cypin overexpression does not significantly change the number of tertiary and high order dendrites. For **(G–I)**, statistics were calculated by unpaired, two-tailed Student's *t*-test. Error bars indicate SEM. *n* = 35 neurons for GFP, and *n* = 51 neurons for cypin.

Cypin overexpression from DIV 10–12 affects dendrites of specific orders, depending on the labeling scheme. For all labeling schemes, the total dendrite count is identical by definition (*p* > 0.9999 as determined by One-Way ANOVA), and cypin overexpression from DIV 10–12 results in an increase in dendrite number that approaches significance (*p* = 0.0581; Figure 6C). The Inside-Out labeling scheme shows that cypin overexpression does not increase primary, secondary, or tertiary and higher order dendrite number (Figures 8A–C). Similarly, the RIT labeling scheme suggests that cypin overexpression does not increase root or intermediate dendrite number (Figure 8D and Figure 8E, respectively). However, cypin overexpression causes an increase in terminal dendrite number that approaches significance (*p* = 0.0747; Figure 8F). Complementing these results, the Tips-In labeling scheme shows that cypin overexpression results in a significant increase in primary dendrites (Figure 8G), which are terminal dendrites or dendrites with no branches (Figure 1, right panel). This significant increase was masked in the previous labeling schemes due to how the dendrites are grouped. Additionally, cypin overexpression causes an increase that approaches significance (*p* = 0.0503) in secondary branches (Figure 8H), which are the penultimate intermediate branch or root dendrites that have branched once (Figure 1, right panel). Using this method, no increases in tertiary and higher order dendrites are detected (Figure 8I).

### Dendrite length for neurons overexpressing cypin from DIV 10–12

**Figure 9:**
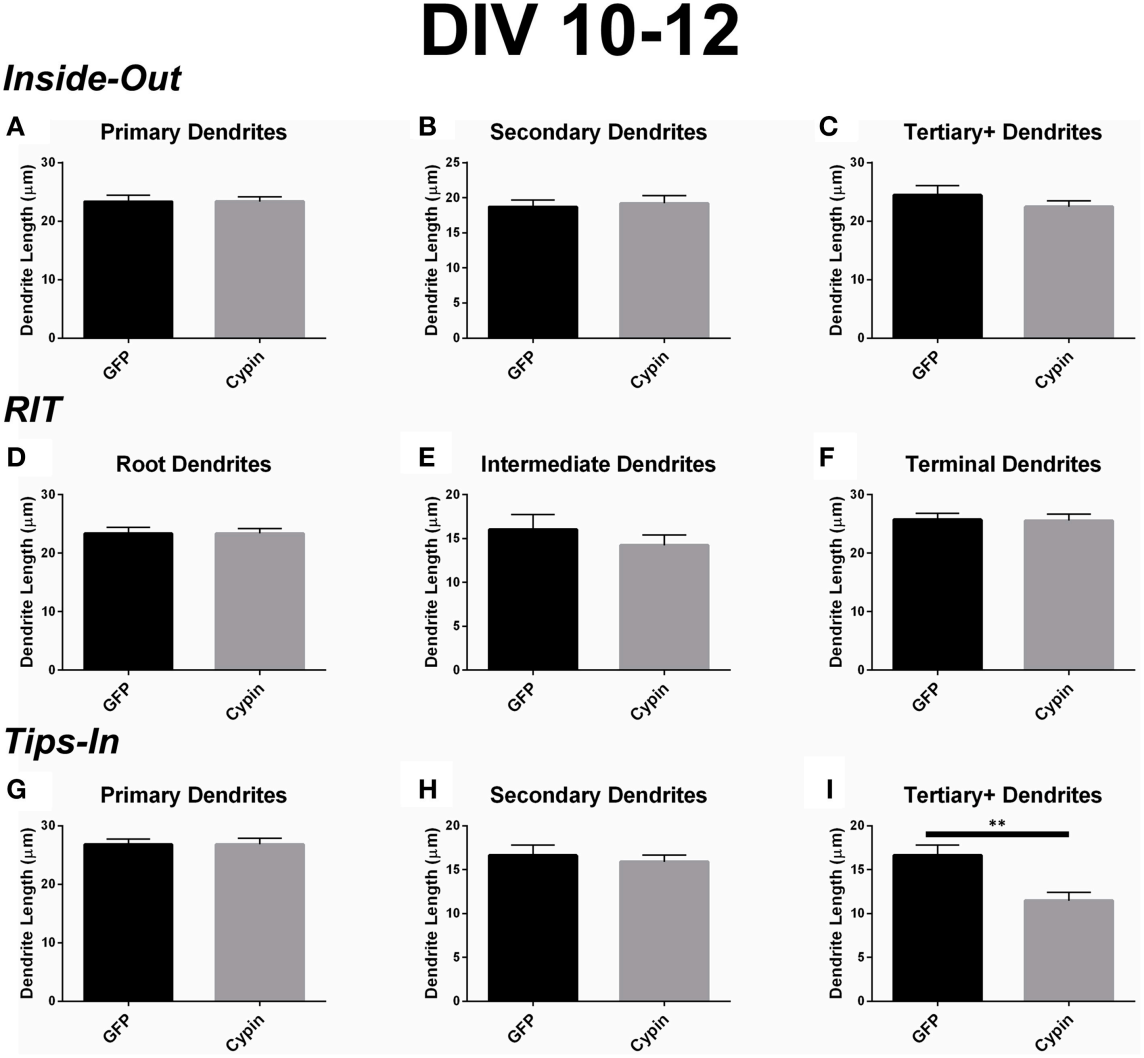
**Cypin decreases the length of innermost dendrites when overexpressed in neurons from DIV 10–12**. **(A–C)** Average dendrite length divided into categories using Inside-Out (conventional) labeling method. **(A)** Cypin overexpression does not significantly change the length of primary dendrites. **(B)** Cypin overexpression does not significantly change the length of secondary dendrites. **(C)** Cypin overexpression does not significantly change the length of tertiary and higher order dendrites. For **(A)**, statistics were calculated by unpaired, two-tailed Student's *t*-tests, and for **(B,C)**, statistics were calculated by unpaired, two-tailed Student's *t*-tests with Welch's correction. **(D–F)** Average dendrite length divided into categories using RIT labeling method. **(D)** Cypin overexpression does not significantly change the length of root dendrites. **(E)** Cypin overexpression does not significantly change the length of intermediate dendrites. **(F)** Cypin overexpression does not significantly change the length of terminal dendrites. For **(D)**, statistics were calculated by unpaired, two-tailed Student's *t*-tests, and for **(E,F)**, statistics were calculated by unpaired, two-tailed Student's *t*-tests with Welch's correction. **(G–I)** Average dendrite length divided into categories using Tips-In labeling method. **(G)** Cypin overexpression does not significantly change the length of primary dendrites. **(H)** Cypin overexpression does not significantly change the length of secondary dendrites. **(I)** Cypin overexpression significantly decreases the length of tertiary and higher order dendrites (^**^p < 0.01). For **(G,H)**, statistics were calculated by unpaired, two-tailed Student's *t*-tests with Welch's correction, and **(I)**, statistics were calculated by unpaired, two-tailed Student's *t*-tests. Error bars indicate SEM. *n* = 35 neurons for GFP, and *n* = 51 neurons for cypin.

Cypin overexpression from DIV 10–12 results in changes to the length of specific orders of dendrites, depending on the labeling scheme. For all labeling schemes, the total dendrite count is identical, and cypin overexpression from DIV 10–12 does not affect overall dendrite length (Figure 6D). The Inside-Out labeling scheme suggests that cypin overexpression does not affect dendrite length according to this labeling method (Figures 9A–C). Similarly, the RIT labeling scheme suggests that cypin overexpression has no effect on dendrite length (Figures 9D–F). In contrast, the Tips-In labeling scheme suggests that cypin overexpression results in a significant decrease in the length of tertiary and higher order dendrites (Figure 9I) without any effect on primary or secondary dendrite length (Figure 9G and Figure 9H, respectively). Taken together, these data suggest that overexpression of cypin specifically affects the lengths of root dendrites with two or more branches. Moreover, we would not have detected this subtle change without using the Tips-In labeling method.

## Discussion

### Developmental effects of cypin overexpression is revealed by three types of sholl analysis

Our laboratory has published a number of studies reporting an important role for the protein cypin in the regulation of dendrite branching and arborization (Akum et al., 2004; Charych et al., 2006; Chen and Firestein, 2007; Fernandez et al., 2008; Kwon et al., 2011). Until now, our data have been presented as changes in primary and secondary dendrite number and analyzed by conventional Sholl analysis. In the present study, we ask whether the combination of different types of Sholl analysis can uncover local changes to the dendritic arbor promoted by cypin overexpression. This work employs our semi-automated Sholl analysis program (“Bonfire”) (Langhammer et al., 2010) and includes three different labeling schemes (Inside-Out, RIT, and Tips-In) to identify previously unreported changes to the dendritic arbor.

To elucidate whether cypin mediates distinct effects on the arbor at different developmental timepoints, we overexpressed cypin from DIV 6–10 and from DIV 10–12, corresponding to periods of active proximal and distal branching (Dotti et al., 1988). When overexpressed from DIV 6–10, cypin increases dendritic branching at 0–42 μm from the soma (Figure 2B), but when overexpressed from DIV 10–12, cypin increases dendritic branching at 18–30 μm from the soma (Figure 6B). Thus, the increase in dendrite branching caused by cypin occurs farther out from the soma at the later developmental time point, suggesting that cypin has specific effects. This may be due to the fact that cypin promotes local microtubule assembly (Akum et al., 2004), and the location of this assembly during distinct times in development, possibly dependent on to where cypin is targeted, may affect specific regions of the arbor.

In terms of dendrite number, cypin significantly increases total dendrite number (Figure 2C) when overexpressed from DIV 6–10 with decreased overall dendrite length (Figure 2D). An increase that approaches significance (*p* = 0.0581) in total dendrite number is observed when cypin is overexpressed from DIV 10–12 (Figure 6C) with no change in average length (Figure 6D). Taken together, these data suggest that cypin alters the dendritic arbor uniquely depending on when in development it is overexpressed. The observed differences in the effect on total dendrite number and average dendrite length could be caused by overexpression of cypin for 96 h versus 48 h. While 48 h (DIV 10–12) was sufficiently long enough to change the dendritic arbor as seen in Total Sholl analysis (Figure 6B), it may not have been long enough to significantly change overall dendrite number in the experiments used for this study.

### The effects of cypin overexpression on the dendritic arbor: Analysis by labeling scheme

Several tools (Sholl, 1953; Van Pelt and Verwer, 1985, 1986; Verwer and Van Pelt, 1990; Caserta et al., 1995; Cannon et al., 1998; Uylings and Van Pelt, 2002; Meijering et al., 2004; Rodriguez et al., 2006) have been developed to assist in Sholl analysis, but to our knowledge, Bonfire is the first to offer multiple labeling schemes and generate individual Sholl graphs of different dendrite categories. We have shown that when using these different labeling schemes—Inside-Out, RIT, and Tips-In—the Sholl curves produced can lead to different interpretations of the effects of cypin overexpression on the process of dendrite branching. Inclusion of all of three of the labeling schemes in analysis provides the most complete picture of changes occurring to the arbor.

While Inside-Out is the traditional method for labeling dendrites, information is lost by grouping tertiary and higher order dendrites together. Depending on how the neuron has developed, there may be many tertiary dendrites proximal to the cell body, and they may be several orders away from the terminal branch that is quite far from the cell body. These two types of dendrites would be grouped together in the Inside-Out labeling scheme. The Root-Intermediate-Terminal (RIT) labeling scheme is better suited for uncovering differences in intermediate and terminal dendrites, as they are in separate categories. The RIT scheme suggests that during DIV 6-10, cypin-promoted increases in intermediate dendrites (Figure 3E) are due to increased secondary dendrites at 6–12 μm from the soma (Figure 3B) and to increased tertiary and higher order dendrites at 30 μm (Figure 3C). Additionally, increased terminal branching (Figure 3F) appears to result in increased tertiary and higher order dendrites due to the corresponding distances at which significant increases occur. However, terminal branching is not responsible for the increases seen at 54–60 μm from the soma for tertiary and higher order dendrites (Figure 3C). For DIV 10–12, the differences in the RIT scheme compared with the Inside-Out scheme indicate that cypin-promoted increases in tertiary and higher order dendrites (Figure 7C) are due to an increase in terminal branches (Figure 7F) at the corresponding distances, which was the same broad similarity observed for DIV 6–10 overexpression. For intermediate dendrites (Figure 7E), it is likely that increased secondary dendrites account for the increased dendrites at 12 μm (Figure 7B), whereas tertiary and higher order dendrites are likely responsible for increased dendrites at 18 μm from the soma (Figure 7C).

The Tips-In labeling scheme reveals subtle differences that are not uncovered by the other two labeling schemes, even when used in combination. Sholl analysis of primary dendrites using the Tips-In method (Figure 3G) suggests that during DIV 6–10, increased proximal dendrites are root dendrites that do not branch, and increased dendrites further from the cell body are higher order terminal branches. For DIV 6–10 overexpression, significant increases are observed at 0–24 μm from the soma for tertiary and higher order dendrites (Figure 3I). The increases at 12 μm and closer to the soma are likely due to primary/root dendrites (Figure 3D), whereas increases further than 12 μm from the soma are likely due to a combination of intermediate dendrites (Figure 3E). For DIV 10–12, increases in Tips-In primary dendrites are observed at 18–30 μm (Figure 7G) due to increased terminal branching (Figure 7F). Unlike changes detected when cypin is overexpressed at DIV 6–10, there are no increases observed at distances that correspond to the root Sholl analysis using the RIT method, indicating that the increases observed in Figure 7G are due to increased terminal branching only and not due to root dendrites. For Tips-In secondary dendrites, increases are observed at 6–18 μm from the soma (Figure 7H). The increase at 6 μm is likely due to primary/root dendrites (Figure 7A), the increase at 12 μm due to secondary dendrites (Figure 7B), and the increase at 18 μm due to tertiary and higher order dendrites (Figure 7C). For tertiary and higher order dendrites (Figure 7I), increases seen within 6 μm from the soma are likely due to root/primary dendrites that have branched twice since no corresponding increases are observed in other dendrite categories.

### Overexpression of cypin promotes shorter higher order dendrites

Regardless of the type of Sholl analysis used, our results strongly suggest that cypin promotes shorter dendrites that are of second order or above. Why would cypin promote increased branching but decreased length? One possibility is that cypin-promoted increases in total dendrites may exhaust a limiting reagent, possibly tubulin or membrane components. A second possibility is that cypin acts via the protein PSD-95. Cypin promotes decreased clustering of PSD-95 (Firestein et al., 1999), which dramatically increases dendrite number but decreases dendrite length (Charych et al., 2006; Sweet et al., 2011a). The de-clustering of PSD-95 may allow correct polarity of microtubules, increasing branching closer to the cell body (Sweet et al., 2011a,b). It is interesting that all labeling schemes yield this result, although the particular orders of dendrites differ. Combining all three Sholl analyses allows us to detect changes to dendrite number and length at subdivisions of the dendritic arbor.

How could cypin-promoted increases in higher order dendrites affect the development of neural networks? Increased number of dendrite branches may allow for increased number of synaptic connections. This may result in increased network activity or, potentially, increased synchronization. Importantly, since these new branches are shorter, local neural circuitry may be affected more than long-range circuitry.

It has been shown by our group that signaling pathways that regulate local changes to the dendritic arbor also act via a cypin-dependent mechanism. For example, brain-derived neurotrophic factor (BDNF) increases cypin protein levels to increase proximal dendritic branching (Kwon et al., 2011). BDNF transcripts may be targeted to the cell body or both the cell body and the dendrites (An et al., 2008), and these distinct trafficking events of BDNF may affect the arbor differently. In addition, RhoA, which itself is locally translated (Wu et al., 2005), regulates the translation of cypin (Chen and Firestein, 2007), which in turn results in increased dendrites. The use of conventional Sholl analysis and dendrite counting may miss the specific effects of local events, and thus, the application of our multiple Sholl analyses will allow for determination of how BDNF and RhoA act to regulate the arbor at specific sites in our future studies.

## Conclusions

**Figure 10:**
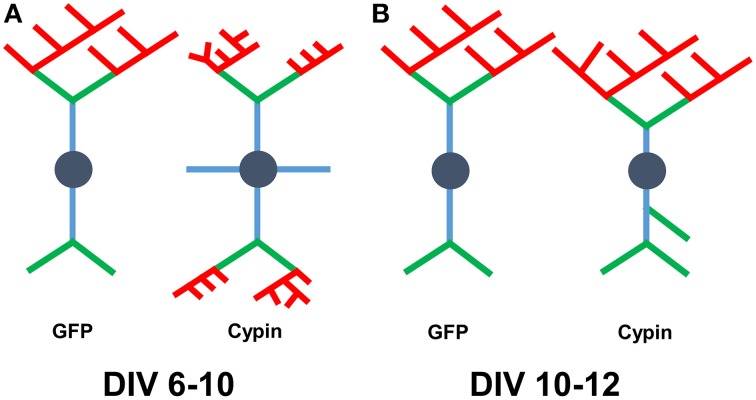
**Schematic of results when combining analysis using all three labeling schemes**. **(A)** Changes that occur to the arbor as a result of cypin overexpression from DIV 6–10. Overexpression of cypin increases primary dendrite numbers (blue) and tertiary and higher order dendrite numbers (red). Overexpression of cypin also increases intermediate dendrite numbers (green and red) and terminal dendrite numbers (green and red) but not secondary dendrite numbers (green). Overexpression of cypin decreases tertiary and higher order dendrite length, intermediate dendrite length, and terminal dendrite length but does not affect primary or secondary dendrite length. **(B)** Changes that occur to the arbor as a result of cypin overexpression from DIV 10–12. Overexpression of cypin increases primary dendrites as labeled by Tips-In (terminal dendrites, green and red) and secondary dendrites as labeled by Tips-In (intermediate or root dendrites, green, and blue). Overexpression of cypin significantly decreases tertiary and higher order dendrite length as labeled by Tips-In (root dendrites that branch twice or more). For schematics shown in **(A,B)**, dendrites are labeled according to the Inside-Out (conventional) method.

Overall, we find that cypin overexpression affects Sholl curves, dendrites numbers, and dendrite lengths differently depending on the developmental timepoint and length of time cypin is overexpressed. Combining our Bonfire program (Langhammer et al., 2010) and these different labeling schemes allows us to better understand how factors, such as cypin, act to regulate neuronal morphology, and hence, function. A schematic summarizing the changes that cypin overexpression exerts on the dendritic arbor is included in Figure 10A for overexpression at DIV 6–10 and in Figure 10B for overexpression at DIV 10–12. Future studies will include collaborations with mathematicians to construct ways to integrate the Inside-Out, RIT, and Tips-In schemes to describe arbors without having to perform the analyses separately. In addition, we would like to use these analyses to devise a system by which we can describe different arbor types (i.e., pyramidal, stellate). Ultimately, we hope that our analyses can be combined with data stored in other neuronal morphology databases (Ascoli et al., 2007). Our ultimate goal is to construct an analysis method that is easy to use, clearly understood, and can serve as a base for comparison between neuron types, different treatments, and experiments performed by different laboratories.

### Conflict of interest statement

Drs. Bonnie L. Firestein reports patent US US7338769 B2 titled “Methods for identifying agonists of cypin” and patent US 7790843 B2 titled “Cypin polypeptide and fragments thereof.”

## References

[B1] Akum B. F. Chen M. Gunderson S. I. Riefler G. M. Scerri-Hansen M. M. Firestein B. L. (2004). Cypin regulates dendrite patterning in hippocampal neurons by promoting microtubule assembly. Nat. Neurosci. 7, 145–152. 10.1038/nn1179 14730308

[B2] An J. J. Gharami K. Liao G. Y. Woo N. H. Lau A. G. Vanevski F.. (2008). Distinct role of long 3′ UTR BDNF mRNA in spine morphology and synaptic plasticity in hippocampal neurons. Cell 134, 175–187. 10.1016/j.cell.2008.05.045 18614020 PMC2527207

[B3] Ascoli G. A. Donohue D. E. Halavi M. (2007). NeuroMorpho.Org: a central resource for neuronal morphologies. J. Neurosci. 27, 9247–9251. 10.1523/JNEUROSCI.2055-07.2007 17728438 PMC6673130

[B4] Cannon R. C. Turner D. A. Pyapali G. K. Wheal H. V. (1998). An on-line archive of reconstructed hippocampal neurons. J. Neurosci. Methods 84, 49–54. 10.1016/S0165-0270(98)00091-0 9821633

[B5] Caserta F. Eldred W. D. Fernandez E. Hausman R. E. Stanford L. R. Bulderev S. V.. (1995). Determination of fractal dimension of physiologically characterized neurons in two and three dimensions. J. Neurosci. Methods 56, 133–144. 10.1016/0165-0270(94)00115-W 7752679

[B6] Charych E. I. Akum B. F. Goldberg J. S. Jornsten R. J. Rongo C. Zheng J. Q.. (2006). Activity-independent regulation of dendrite patterning by postsynaptic density protein PSD-95. J. Neurosci. 26, 10164–10176. 10.1523/JNEUROSCI.2379-06.2006 17021172 PMC6674632

[B7] Chen H. Firestein B. L. (2007). RhoA regulates dendrite branching in hippocampal neurons by decreasing cypin protein levels. J. Neurosci. 27, 8378–8386. 10.1523/JNEUROSCI.0872-07.2007 17670984 PMC6673065

[B8] Dong X. Shen K. Bülow H. E. (2015). Intrinsic and extrinsic mechanisms of dendritic morphogenesis. Annu. Rev. Physiol. 77, 271–300. 10.1146/annurev-physiol-021014-071746 25386991

[B9] Dotti C. G. Sullivan C. A. Banker G. A. (1988). The establishment of polarity by hippocampal neurons in culture. J. Neurosci. 8, 1454–1468. 3282038 10.1523/JNEUROSCI.08-04-01454.1988 PMC6569279

[B10] Eilers J. Konnerth A. (1997). Dendritic signal integration. Curr. Opin. Neurobiol. 7, 385–390. 10.1016/S0959-4388(97)80067-0 9232799

[B11] Elston G. N. Fujita I. (2014). Pyramidal cell development: postnatal spinogenesis, dendritic growth, axon growth, and electrophysiology. Front. Neuroanat. 8:78. 10.3389/fnana.2014.00078 25161611 PMC4130200

[B12] Fernández J. R. Welsh W. J. Firestein B. L. (2008). Structural characterization of the zinc binding domain in cytosolic PSD-95 interactor (cypin): role of zinc binding in guanine deamination and dendrite branching. Proteins 70, 873–881. 10.1002/prot.21683 17803218 PMC2721013

[B13] Firestein B. L. Brenman J. E. Aoki C. Sanchez-Perez A. M. El-Husseini A. E.. (1999). Cypin: a cytosolic regulator of PSD-95 postsynaptic targeting. Neuron 24, 659–672. 10.1016/S0896-6273(00)81120-4 10595517

[B14] Häusser M. Spruston N. Stuart G. J. (2000). Diversity and dynamics of dendritic signaling. Science 290, 739–744. 10.1126/science.290.5492.739 11052929

[B15] Kulkarni V. A. Firestein B. L. (2012). The dendritic tree and brain disorders. Mol. Cell. Neurosci. 50, 10–20. 10.1016/j.mcn.2012.03.005 22465229

[B16] Kutzing M. K. Langhammer C. G. Luo V. Lakdawala H. Firestein B. L. (2010). Automated Sholl analysis of digitized neuronal morphology at multiple scales. J. Visual. Exp. 45:2354. 10.3791/2354 21113115 PMC3159598

[B17] Kwon M. Fernández J. R. Zegarek G. F. Lo S. B. Firestein B. L. (2011). BDNF-promoted increases in proximal dendrites occur via CREB-dependent transcriptional regulation of cypin. J. Neurosci. 31, 9735–9745. 10.1523/JNEUROSCI.6785-10.2011 21715638 PMC3139247

[B18] Landgraf M. Evers J. F. (2005). Control of dendritic diversity. Curr. Opin. Cell Biol. 17, 690–696. 10.1016/j.ceb.2005.09.005 16226445

[B19] Langhammer C. G. Previtera M. L. Sweet E. S. Sran S. S. Chen M. Firestein B. L. (2010). Automated Sholl analysis of digitized neuronal morphology at multiple scales: whole cell Sholl analysis versus Sholl analysis of arbor subregions. Cytometry Part A 77A, 1160–1168. 10.1002/cyto.a.20954 20687200 PMC4619108

[B20] Libersat F. (2005). Maturation of dendritic architecture: lessons from insect identified neurons. J. Neurobiol. 64, 11–23. 10.1002/neu.20142 15884008

[B21] Meijering E. Jacob M. Sarria J. C. Steiner P. Hirling H. Unser M. (2004). Design and validation of a tool for neurite tracing and analysis in fluorescence microscopy images. Cytometry Part A 58A, 167–176. 10.1002/cyto.a.20022 15057970

[B22] Miller J. P. Jacobs G. A. (1984). Relationships between neuronal structure and function. J. Exp. Biol. 112, 129–145. 6392465 10.1242/jeb.112.1.129

[B23] Rodriguez A. (2007). NeuronStudio Documentation. Available online at: http://research.mssm.edu/cnic/help/ns/labeledges.html

[B24] Rodriguez A. Ehlenberger D. B. Hof P. R. Wearne S. L. (2006). Rayburst sampling, an algorithm for automated three-dimensional shape analysis from laser scanning microscopy images. Nat. Protoc. 1, 2152–2161. 10.1038/nprot.2006.313 17487207

[B25] Sainath R. Gallo G. (2015). Cytoskeletal and signaling mechanisms of neurite formation. Cell Tissue Res. 359, 267–278. 10.1007/s00441-014-1955-0 25080065 PMC4286448

[B26] Santiago C. Bashaw G. J. (2014). Transcription factors and effectors that regulate neuronal morphology. Development 141, 4667–4680. 10.1242/dev.110817 25468936 PMC4299270

[B27] Schaefer A. T. Larkum M. E. Sakmann B. Roth A. (2003). Coincidence detection in pyramidal neurons is tuned by their dendritic branching pattern. J. Neurophysiol. 89, 3143–3154. 10.1152/jn.00046.2003 12612010

[B28] Sholl D. A. (1953). Dendritic organization in the neurons of the visual and motor cortices of the cat. J. Anat. 87, 387–406. 13117757 PMC1244622

[B29] Sweet E. S. Previtera M. L. Fernández J. R. Charych E. I. Tseng C. Y. Kwon M.. (2011a). PSD-95 alters microtubule dynamics via an association with EB3. J. Neurosci. 31, 1038–1047. 10.1523/JNEUROSCI.1205-10.2011 21248129 PMC3138189

[B30] Sweet E. S. Tseng C. Y. Firestein B. L. (2011b). To branch or not to branch: how PSD-95 regulates dendrites and spines. Bioarchitecture 1, 69–73. 10.4161/bioa.1.2.15469 21866266 PMC3158629

[B31] Uylings H. B. Van Pelt J. (2002). Measures for quantifying dendritic arborizations. Network 13, 397–414. 10.1088/0954-898X/13/3/309 12222821

[B32] Van Pelt J. Verwer R. W. (1985). Growth models (including terminal and segmental branching) for topological binary trees. Bull. Math. Biol. 47, 323–336. 10.1007/BF02459919 4041665

[B33] Van Pelt J. Verwer R. W. (1986). Topological properties of binary trees grown with order-dependent branching probabilities. Bull. Math. Biol. 48, 197–211. 10.1007/BF02460023 3719156

[B34] Verwer R. W. Van Pelt J. (1990). Analysis of binary trees when occasional multifurcations can be considered as aggregates of bifurcations. Bull. Math. Biol. 52, 629–641. 10.1007/BF02462102 2224283

[B35] Vetter P. Roth A. Hausser M. (2001). Propagation of action potentials in dendrites depends on dendritic morphology. J. Neurophysiol. 85, 926–937. 11160523 10.1152/jn.2001.85.2.926

[B36] Wu K. Y. Hengst U. Cox L. J. Macosko E. Z. Jeromin A. Urquhart E. R.. (2005). Local translation of RhoA regulates growth cone collapse. Nature 436, 1020–1024. 10.1038/nature03885 16107849 PMC1317112

[B37] Zoghbi H. Y. (2003). Postnatal neurodevelopmental disorders: meeting at the synapse? Science 302, 826–830. 10.1126/science.1089071 14593168

